# γδT Cells Are Prevalent in the Proximal Aorta and Drive Nascent Atherosclerotic Lesion Progression and Neutrophilia in Hypercholesterolemic Mice

**DOI:** 10.1371/journal.pone.0109416

**Published:** 2014-10-14

**Authors:** Duc M. Vu, Albert Tai, Jeffrey B. Tatro, Richard H. Karas, Brigitte T. Huber, Debbie Beasley

**Affiliations:** 1 Molecular Cardiology Research Institute, Tufts Medical Center, Boston, Massachusetts, United States of America; 2 Department of Integrative Physiology and Pathobiology, Tufts University School of Medicine, Boston, Massachusetts, United States of America; 3 Division of Endocrinology, Diabetes and Metabolism, Tufts Medical Center, Boston, Massachusetts, United States of America; University of Amsterdam Academic Medical Center, Netherlands

## Abstract

Unique innate immunity-linked γδT cells have been seen in early human artery lesions, but their role in lesion development has received little attention. Here we investigated whether γδT cells modulate atherogenesis in apolipoprotein E-deficient (ApoE KO) mice. We found that γδT cell numbers were markedly increased in the proximal aorta of ApoE-deficient *vs.* wild-type mice during early atherogenesis, particularly in the aortic root and arch, where they comprised most of the T cells and lesion progression is most rapid. γδT cells infiltrated intimal lesions in ApoE KO mice, but only the adventitia in wild-type mice, and were more prevalent than CD4^+^ T cells in early nascent lesions, as evaluated by *en face* confocal microscopy. These aortic γδT cells produced IL-17, but not IFN-γ, analyzed by *ex vivo* FACS. Furthermore, aortic arch lipid accumulation correlated strongly with abundance of IL-17-expressing splenic γδT cells in individual ApoE KO mice. To investigate the role of these γδT cells in early atherogenesis, we analyzed ApoE/γδT double knockout (DKO) compared to ApoE KO mice. We observed reduced early intimal lipid accumulation at sites of nascent lesion formation, both in chow-fed (by 40%) and Western diet-fed (by 44%) ApoE/γδT DKO mice. In addition, circulating neutrophils were drastically reduced in these DKO mice on Western diet, while expansion of inflammatory monocytes and splenic Th1 or Th17 lymphocytes was not affected. These data reveal, for the first time, a pathogenic role of γδT cells in early atherogenesis in ApoE KO mice, by mechanisms likely to involve their IL-17 production and induction of neutrophilia. Targeting γδT cells thus might offer therapeutic benefit in atherosclerosis or other inflammatory vascular diseases.

## Introduction

Atherosclerosis is a chronic inflammatory disease of the inner lining of large- and medium-sized arteries and a leading cause of cardiovascular disease and mortality worldwide. Converging evidence points to a role of adaptive immunity and T cell subsets, including T helper 1 (Th1), Th2, Th17, and regulatory T cell subsets, in human and mouse atherogenesis [1]–[4], deduced primarily by defining the roles of the prototypical cytokines that each produces. A proatherogenic role of Th1 cells is supported by findings that exogenous IFN-γ promotes atherogenesis [5] and mice lacking IFN-γ [6], [7], the IFN-γ receptor [8], or the Th1 cell transcription factor T-bet [9] are resistant to atherosclerosis. More recently, a proatherogenic role of IL-17-expressing Th17 cells has been posited based on evidence of increased lesion size and leukocyte content in ApoE knockout (KO) mice receiving exogenous IL-17 [10], and reduced lesion size and leukocyte content in ApoE KO mice with IL-17 or IL-17 receptor deficiency [10]–[14]. Also, IL-17-expressing cells are found in the aortic root in a mouse model of human familial hypercholesterolemia, and oxidized LDL can stimulate dendritic cell-dependent Th17 cell polarization *in vitro* [15].

T lymphocytes can also be divided into 2 subsets based on the T cell receptor (TCR) chains that they express, αβ or γδ and notably both subsets can produce IFN-γ and IL-17 [16]. The majority of T cells in peripheral blood and lymphoid tissues express TCRαβ and mediate adaptive immunity when activated by antigenic peptides that are recognized in the context of MHC molecules. However, a minor subset of T cells expresses TCRγδ, has limited TCR diversity, and is enriched at epithelial and mucosal surfaces where these cells respond rapidly to pathogens and promote innate immunity [17]. Most T cells in human arterial lesions express TCRαβ, but some express TCRγδ. Such γδT cells are enriched in fatty streaks and the transition zone between normal intima and atherosclerotic plaque, comprising up to 20% of T cells [18], [19], but are rarely found in the normal intima of children [20], pointing to the possibility that γδT cells regulate early atherogenesis [21]. Recently, IL-17-expressing γδT cells (γδT17), together with Th17 cells, were found in aorta of ApoE KO mice [11], but their respective roles in atherogenesis are unknown. An earlier study found a stronger role of αβT cells in early atherogenesis in female ApoE KO mice, because TCRβ deficiency reduced lesion area by 50%, whereas the effect of TCRδ deficiency was more modest (21%) and not statistical significant [22], [23], pointing to a potential supporting role of γδT cells. Recently, it has been proposed that hypercholesterolemia activates γδT cell signaling and proliferation [24]; thus, the potential role of γδT cells in atherogenesis merits further assessment. Here we tested the role of γδT cells in early atherogenesis in male ApoE KO mice. Because lesion progression is site-specific [22], we compared lesion areas in the aortic root, arch and descending aorta of ApoE KO *vs.* ApoE/TCRδ double-KO (DKO) mice and determined whether γδT cells infiltrated early lesions at these sites.

Remarkably, we found that γδT cells are the major T cell subset in early aortic root and arch lesions in ApoE KO mice, and many of these are γδT17 cells. Moreover, our findings with ApoE/γδT DKO mice point to a role of γδT cells in promoting nascent lesion progression and neutrophilia during early atherogenesis.

## Materials and Methods

### Generation of ApoE and γδT cell double knockout mice

ApoE KO [25] and TCRδ KO [26] mice were obtained from Jackson Laboratories. Both strains of mice have been backcrossed 12 times on the C57BL/6 background, thus any differences in atherosclerotic lesion formation should be due to targeted deletions rather than to insufficient backcrossing of donor mice. DKO mice were obtained by breeding ApoE KO male mice to TCRδ KO female mice, and then intercrossing the heterozygous littermate mice to obtain the DKO genotype, as determined by PCR analysis of ear-punch DNA and FACS analysis of blood for the presence of γδT cells. No significant differences were observed in the overall health or behavior of ApoE KO *vs*. γδT DKO mice. All animal procedures were approved by the Tufts Institutional Animal Care and Use Committee.

### Aortic lipid accumulation

Early aortic lipid accumulation was studied in two groups of male mice, 24 wk-old mice fed chow, and 14 wk-old fed Western diet (TD.88137; Harlan Teklad) for 4 weeks. Aortic lipid was analyzed in chow-fed mice by *en face* analysis as previously described [27]. Mice were sacrificed by CO_2_ inhalation, perfused with PBS containing 20 U/ml sodium heparin via the left ventricle, and the aortic root, arch, and descending aorta were removed and fixed 2 h at room temperature with 4% paraformaldehyde. Neutral lipid was visualized by staining with Oil Red O solution (ORO; 0.5% in 60% isopropanol). Aortic root, aortic arch and descending aorta segments were flattened on separate glass slides with glass coverslips and mounting media (Aqua Mount; Thermo Scientific). Digital images were obtained using a dissecting microscope and digital camera, and analyzed using ImageJ software (NIH).

Western diet-fed mice were perfused as described above, and the aortic arch and descending aorta analyzed *en face*. The aortic root and associated heart tissue were embedded in OCT, stored at −80°C, then sectioned with a cryostat. Sections (10 µm each) were collected form each aortic root, beginning at the aortic sinus when 3 valve leaflets became visible, such that each glass slide contained 4 sections at 80 µm intervals throughout the entire aortic root. Two slides were stained with ORO and hematoxylin to visualize neutral lipid; 2 additional slides were stained with anti-CD68 FITC to visualize macrophages which comprise most of the lesion at this early stage, and DAPI and Alexa Fluor 633 to visualize nuclei and elastin. Digital images were obtained with a Nikon epifluorescence microscope, and lesions were quantitated with ImageJ software. Lesion areas are presented as the average of 4 sections spanning 240 µm and showing 3 complete cusps.

### Serum cholesterol and triglycerides

Total cholesterol and triglycerides in serum were analyzed using enzymatic methods and commercial kits and standards (Pointe Scientific, Inc.).

### Proximal aorta cell suspensions

Aortas were dissected from the aortic root to the top of the diaphragm after perfusion of the vasculature, and cleared of adventitial fat. Single cell suspensions were obtained by digesting the tissue for 1 h at 37°C in PBS with 450 U/ml collagenase type I, 125 U/ml collagenase Type XI, 60 U/ml hyaluronidase type I-S, and 60 U/ml DNase I (all from Sigma), as previously described [28]. Digests were filtered thru a 70 µm filter and resuspended in FACS buffer.

### FACS analysis of aortic T lymphocytes

Aortic cells were incubated with anti-CD16/CD32 to block Fc receptors (10 min), then 30 min with anti-CD3e-FITC (145-2C11), anti-CD45-PE (30-F11) and anti-γδTCR APC (GL3) (all from eBioscience). CountBright absolute counting beads (Invitrogen) were added to each sample immediately before analysis using a BD FACSCALIBUR with 4-color capacity in the Tufts Pathology Flow Cytometry Core facility. The number of fluorescent beads acquired during each FACS run (gating on the characteristic low forward scatter/high side scatter and high FL3 fluorescence events) was used to calculate the total aortic cell numbers. γδT cells detected in aortic tissue were not due to residual blood contamination as they were highly enriched (15–90% of total CD3^+^ T cells) relative to their levels in blood (∼2.5% of total CD3^+^ T cells).

In each experiment, populations of fresh splenocytes were single-stained with each individual marker in the panel in order to adjust channel voltages to obtain optimal separation of positive and negative cell populations, and to manually apply compensation adjustments such that single markers did not appear in the other 3 channels, taking care to either apply appropriate compensation, or to undercompensate, but never to overcompensate. Summit 4.3 Software was used to analyze FACS data, and to apply additional compensation as needed. To eliminate cell debris and analyze the aortic lymphocyte population, we applied the gate encompassing the lymphocyte population in fixed and stained splenocytes to the FSC *vs.* SSC plots of aortic cells. Aortic lymphocytes were then further gated on CD45^+^ cells. Specificity of staining was confirmed by additional aliquots of aortic cells stained with isotype control antibodies (***Fig. S1B***), and in the case of γδT cell identification, by the comparison of aortic digests from TCRδ KO mice.

### *En face* analysis of aortic T cells

Adventitial and intimal T cells in aortic root, arch, and thoracic descending aorta were analyzed by *en face* confocal microscopy, as previously described [27]. Mice were perfused with PBS and heparin, and aortic segments harvested, opened longitudinally to expose the lumen, and post-fixed for 30 min at 4°C in 2% paraformaldehyde. Periadventitial fat was removed, and tissues were blocked for 1 h with anti-CD16/CD32 Fc-block (BD Biosciences) and 10 µg/ml non-immune mouse IgG (Sigma). DAPI, anti-CD3e-FITC, and either anti-γδTCR APC or anti-CD4 APC (RM4-5) were added directly to the blocking solution, and the tissues incubated overnight at 4°C. Tissues were then washed with PBS, mounted between two glass coverslips with SlowFade Gold (Molecular Probes), and imaged using a Leica SP2 confocal microscope. T cells were located by scanning the aortic tissue by eye for green fluorescence in the FITC filter, which was easily distinguishable from yellow non-specific autofluorescence. To assess the specificity of far-red fluorescent images obtained with the confocal microscope, we verified that a corresponding orange-red signal was not seen in the fluorescence microscope using the Cy3 HQ filter cube.

### FACS analysis of circulating leukocytes

Erythrocytes were lysed by incubating blood in Pharm-lyse (BD Bioscience) 30 min at room temperature. Blood leukocytes were resuspended in FACS buffer, incubated with anti-Ly6G-FITC (1A8), anti-Ly6B.2(7/4)-PE, anti-CD11b-PERCP/Cy5 (M1/70), and anti-Ly6C-APC (AL-21). All antibodies were from BD Pharmingen except anti-Ly6B.2, which was from Serotec. Cells were washed, resuspended in FACS buffer and analyzed immediately after addition of CountBright counting beads. Plots shown have been gated on the viable leukocyte population in FSC *vs.* SSC plots. Neutrophils were identified as Ly6G^+^ Ly6B^+^. Inflammatory monocytes were identified using a combination of 4 characteristic surface markers as Ly6C^hi^ Ly6G^−^ Ly6B^hi^ CD11b^+^ [29], [30].

### Analysis of IL-17 expression by *ex vivo* stimulation and intracellular staining

Splenocytes were dispersed through a 70 µm cell strainer with RPMI, and erythrocytes removed by incubation in Pharm-Lyse (30 min; BD Bioscience). Aortic cells and splenocytes were resuspended in RPMI supplemented with 10% FCS, sodium pyruvate, penicillin, streptomycin and β-mercaptoethanol. Cells were incubated 4 h at 37°C after adding PMA (20 ng/ml) and ionomycin (400 ng/ml) to boost the level of ongoing IL-17 production and GolgiPlug (BD Bioscience) to retain IL-17 intracellularly. Cells were then harvested, washed, incubated with anti-CD3e-FITC and either anti-γδTCR APC or anti-CD4 APC, fixed with 4% paraformaldehyde (10 min), permeabilized with saponin (1 mg/ml), incubated 30 min with anti-IL-17-PE (eBio17B7), anti-IFN-γ-PE (XMG1.2) or isotype control antibody-PE, washed, and analyzed by FACS. After gating on the viable lymphocyte population, IL-17^+^ cells within CD3^+^ TCRγδ^+^ and CD3^+^CD4^+^ cell populations (γδT17 and Th17 cells, respectively) were analyzed. To calculate IL-17-specific staining, the % cells staining positive with isotype control antibody (***Fig. S1c***) was subtracted from the % staining positive with anti-IL-17.

### Statistical analysis

Significant differences between 2 groups were determined by unpaired t tests, and differences between 3 or more groups were determined by Kruskall-Wallis followed by Dunn’s multiple comparison test when group variances were unequal, or ANOVA followed by Bonferonni’s multiple comparisons test using Prism Graphpad software. Values of *p*<0.05 were considered significant.

## Results

### γδT cells are elevated in the proximal aorta of ApoE KO mice

**Figure 1:**
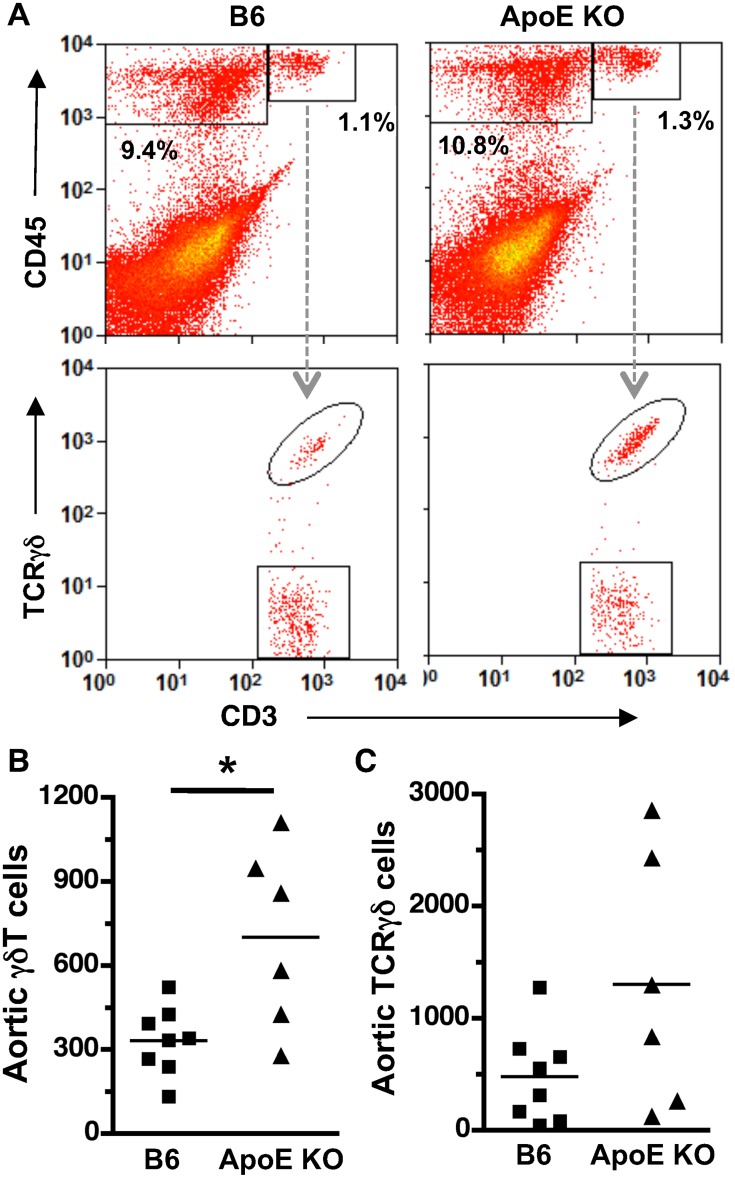
Increased γδT cells in proximal aorta of ApoE KO vs. B6 mice. Single-cell suspensions from enzyme-digested aortas of 22 wk-old chow-fed mice were stained with anti-CD3-FITC, anti-CD45-PE and anti-TCRγδ-APC and analyzed by FACS. **A:** Gating strategy and representative FACS plots. **B:** *Bar*s indicate means; *symbols* indicate the absolute number of aortic T cells in individual mice. γδT cells (CD3^+^TCRγδ^+^) were significantly increased in ApoE KO *vs.* B6 aorta (*p<0.04). The absolute numbers of conventional αβT cells (CD3^+^TCRγδ^−^) were highly variable in individual aortas from ApoE KO mice and were not significantly different from the numbers in B6 mice.

Using FACS analysis, we compared the total numbers of γδT cells in proximal aorta, where early lesions develop in 22 wk-old chow-fed ApoE KO mice, *vs.* those in the lesion-free proximal aortas of wild-type C57BL6 (B6) mice. Leukocytes (CD45^+^) and T cells (CD45^+^CD3^+^) were readily distinguished from vascular wall cells (CD45^−^), and the conventional αβT cell (CD3^+^ TCRγδ^−^) and γδT cell (CD3^+^ TCRγδ^+^) populations were clearly distinct (**Fig. 1** **,** ***S1***). Aortic γδT cell numbers were increased 2.5-fold in ApoE KO *vs*. B6 mice (*p<0.04*), whereas conventional T cell numbers were variable and showed no significant increase. Therefore, ApoE deficiency is associated with expanded γδT cell populations in the lesion-susceptible proximal aorta, suggesting a potential role of γδT cells in early disease progression.

### γδT cells infiltrate early aortic root and arch lesions and adjacent adventitia in ApoE KO mice

**Figure 2:**
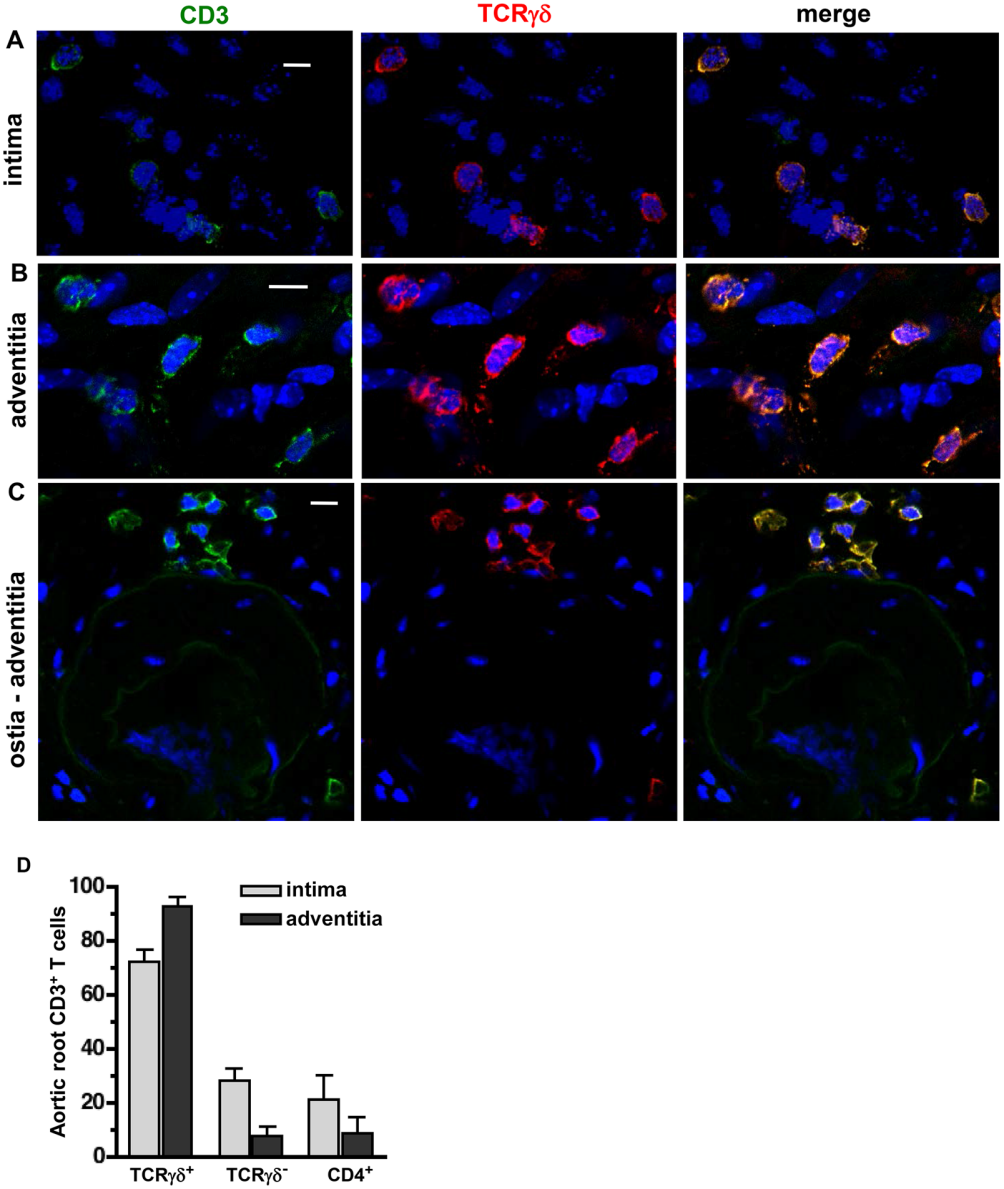
γδT cells are the dominant T cell subset in early lesions and associated adventitia of ApoE KO mice. *En face* confocal images of aortic tissue from Western diet-fed ApoE KO mice stained with anti-CD3-FITC (*green*), anti-TCRγδ APC (*red*), and DAPI *(blue)*. γδT cells constitute the majority of CD3^+^ T cells in aortic root intima (***A***) and adventitia (***B***). γδT cells infiltrate the adventitia at intercostal artery branch points of the descending thoracic aorta (***C***). White scale bar = 10 µm. **D:** Quantitation: n = 3.

Considering the marked enrichment of γδT cells in proximal aorta of ApoE KO *vs.* B6 mice, we determined the precise location of T cells within intima and adventitia, by *en face* confocal immunostaining. γδTcells (CD3^+^TCRγδ^+^) were clearly distinguishable from conventional αβT cells (CD3^+^TCRγδ^−^) cells (***Fig. S2***), and were absent from aortas of ApoE/TCRδ DKO mice (*not shown*), confirming the specificity of the staining. Remarkably, most aortic root T cells associated with early lesions were γδT cells, constituting 72% of intimal T cells and 97% of adventitial T cells of Western diet-fed ApoE KO mice (**Fig. 2A, B, D**). In contrast, both CD4^+^ and CD3^+^TCRγδ^−^ cells constituted only ∼20% of early intimal T cells (**Fig. 2D** **,** ***S3***), and were rare in the adventitia. No γδT cells were found to express CD4 (***Fig. S3***). γδT cells, although more prevalent in the aortic root, were also found in aortic arch lesions and adjacent adventitia (not shown). In the thoracic descending aorta, γδT cells were not found in the intima, and within the adventitia they were only found at intercostal artery branch points (**Fig. 2C**), sites prone to lesion formation [31]. In wild-type mice, γδT cells were less abundant, found mostly in adventitia at the base of the aortic root, but only rarely in intima (not shown). γδT cells were also present in advanced lesions of aged chow-fed ApoE KO mice, but CD4^+^ T cells were somewhat more prevalent at this stage (***Fig. S4***). These results show that γδT cells are present in early stage atherosclerotic lesions of the aortic root and arch of ApoE KO mice.

*En face* microscopic analysis showed consistent predominance of γδT cells in intima and adventitia (72% and 97%, **Fig. 2D**). In contrast, FACS analysis of enzyme-dispersed aortic cells showed highly variable numbers of conventional T cells (**Fig. 1C**), and thus γδT cells expressed as a percentage of total T cells was highly variable, from 15%–90%, and not significantly different between B6 *vs.* ApoE KO mouse aorta (50±11% *vs.* 45±10%). It is possible that the variable prevalence of conventional T cells in enzyme-dispersed aortic cell populations is due to contaminating remnants of lymphatic vessels [32] or periadventitial fat [33]. Lymphatic or adipose-associated cells were not included in the cell counts when T cells were identified visibly by confocal microscopy (**Fig. 2A–C**).

### γδT17 cells are increased in aorta and spleen of ApoE KO mice

**Figure 3:**
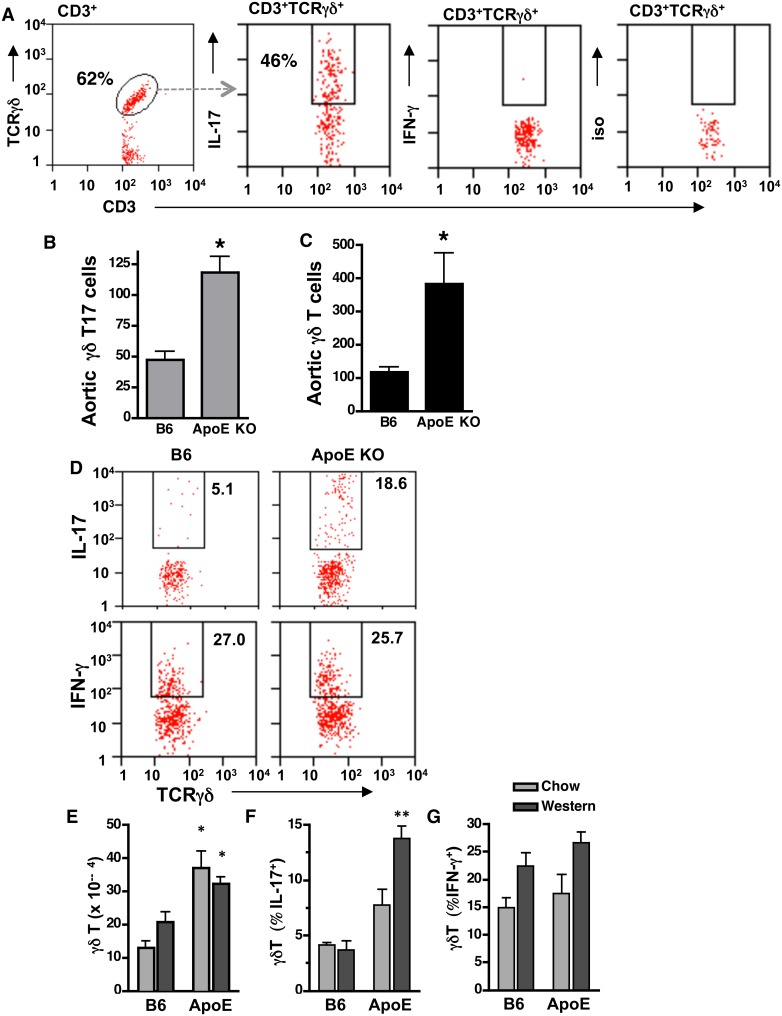
Increased aortic and splenic γδT17 cells in ApoE KO mice. Proximal aorta and spleen were isolated from 14 wk-old B6 and ApoE KO mice. Aortic cells (**A–C**) and splenocytes (**D–G**) were analyzed by FACS after staining for cell surface TCRγδ and intracellular IL-17 or IFN-γ. **A:** Representative FACS plots of Western diet-fed ApoE KO mouse aortic cells, gated on the cell populations indicated at the top of the plot. **B&C:** Total aortic γδT17 and γδT cells are increased in Western diet-fed ApoE KO (n = 10) *vs.* B6 (n = 9) mice (*p<0.02). **D:** Representative FACS plots of splenocytes in Western diet-fed ApoE KO mice. **E:** Total splenic γδT cells are increased in ApoE KO *vs.* diet-matched B6 mice. **F&G:** IL-17, but not IFN-γ, expression is increased in splenic γδT cells of Western diet-fed ApoE KO (n = 14) *vs.* B6 mice (n = 9). *p<0.05, **p<0.01 ApoE KO *vs.* diet-matched B6 mice.

To determine whether aortic γδT cells produce IL-17, we isolated intimal and adventitial leukocytes by enzymatic digestion and analyzed them by intracellular FACS. Aortic γδT17 cell numbers were markedly increased in Western diet-fed ApoE KO *vs.* B6 mice (**Fig. 3A, B**). This was due to increased total numbers of aortic γδT cells (**Fig. 3C**), as the percentage of aortic γδT cells that were expressing IL-17 was similar in both groups (29±4% in B6 *vs.* 29±3% in ApoE KO). Aortic γδT cells did not produce IFN-γ (**Fig. 3A**).

Western diet feeding also induced significant increases in splenic γδT17 cells in ApoE KO *vs.* B6 mice, which was due to both increased total splenic γδT cells (**Fig. 3E**) and increased % expressing IL-17 (**Fig. 3D, F**). In contrast, Western diet feeding induced only a modest, non-significant increase in IFN-γ expression in splenic γδT cells of ApoE KO mice (**Fig. 3G**), and γδT cell IFN-γ expression was similar in wild-type mice that do not develop hypercholesterolemia or aortic lesions after Western diet feeding (**Table 1**). Together the results indicate that Western diet-induced hypercholesterolemia specifically expands aortic and splenic γδT17 cells in ApoE KO mice.

**Table 1:** Body and spleen weights, and serum lipids in chow- *vs.* Western diet-fed B6 and ApoE KO mice.

Mouse	Diet	Body wt (g)	Spleen wt (mg)	Cholesterol (mg/dl)	Triglyceride (mg/dl)	n
B6	Chow	29.0±1.3	75±5	181±28	104±1	4
B6	Western	35.7±0.9*	92±5	288±63	118±16	9
ApoE KO	Chow	30.1±0.3	102±4*	395±68*	180±38	4
ApoE KO	Western	36.6±0.6^#^	105±5*	1204±68*	246±21*	10

Values are mean ± SE. *P<0.02 *vs*. B6 chow. ^#^ *P*<0.02 *vs*. ApoE KO chow.

Splenic T cell populations for these mice are shown in Fig. 3D–G and 7.

### Aortic arch lesion area and splenic Th17 cells correlate with splenic γδT17 cell expansion in Western diet-fed ApoE KO mice

To assess the potential relationships between hyperlipidemia, Western diet-induced increases in IL-17-expressing T cell subpopulations, and aortic lipid accumulation, we tested whether they were significant correlations of these parameters in individual mice. We found that aortic arch lesion area correlated strongly with IL-17 expression in splenic γδT cells in Western diet-fed ApoE KO mice (**Fig. S5**), suggesting a role of γδT17 cells in aortic arch disease. Splenic Th17 cell numbers were also significantly correlated with splenic γδT cells (***Fig. S5B***), suggesting a potential connection between these two IL-17-expressing cell populations. Because hypercholesterolemia activates γδT cell signaling and proliferation [24], we tested for correlations between serum lipids and splenic γδT17 cells. Surprisingly, splenic γδT17 cell numbers were negatively correlated with serum cholesterol levels in individual mice (***Fig. S5C***), and were not correlated with serum triglyceride (***Fig. S5D***). The latter negative correlation may be related to the fact serum cholesterol levels are quite high in ApoE KO mice consuming Western diet.

### γδT cell deficiency reduces early aortic lesion in the aortic root and arch of ApoE KO mice

**Figure 4:**
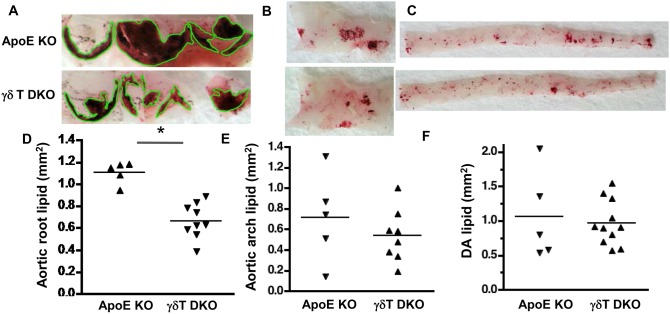
γδT cell deficiency reduces aortic root lesion area in 24 wk-old chow-fed ApoE KO mice. **A&D:** Aortic root lipid-rich lesions outlined in *green* (*en face* view) are significantly decreased in ApoE/γδT DKO mice (*p*<0.0001). Lesion area was not affected by γδT cell deficiency in the aortic arch (**B&E**) or descending aorta (**C&F**).

To determine whether γδT cells affect lesion development in the aortic root or arch, we generated γδT cell-deficient ApoE KO mice (DKO). γδT cells were not detectable by FACS analysis in blood, aorta, spleen or lymph nodes of chow-fed ApoE/TCRδ DKO mice (***Fig. S1A*** and not shown), confirming the γδT cell-deficient phenotype. γδT cell-deficiency reduced lipid-rich *en face* lesion area by 40% in the aortic root (**Fig. 4A** **;** p<0.0001), the site of most active lesion progression in 24 wk-old chow-fed mice. Lesions were infrequent in aortic arch and descending aorta at this early age and stage of disease, and effects of γδT cell deficiency were not detected (**Fig. 4B&C**). γδT cell deficiency did not affect body weight, spleen weight, or serum triglyceride, but did increase total serum cholesterol in chow-fed ApoE KO mice (**Table 2**), indicating that γδT cell-deficiency reduced aortic root lesions in chow-fed mice, despite the concurrent increase in serum cholesterol.

**Table 2:** Body and spleen weights, serum lipids and SAA levels in ApoE *vs.* ApoE/TCRδ DKO mice.

Mouse	Diet	Body wt (g)	Spleen wt (mg)	Cholesterol (mg/dl)	Triglyceride (mg/dl)	SAA (mg/dl)	n
ApoE KO	Chow	31.4±0.6	97±3	391±40	135±20		5
γδT DKO	Chow	32.8±0.4	99±4	523±28*	147±9		11
ApoE KO	Western	32.8±0.8	109±5	1195±63	243±19	59±6	14
γδT DKO	Western	32.3±1.8	106±6	1232±124	252±30	52±6	9

Values are mean ± SE. *P<0.02 *vs*. ApoE KO.

**Figure 5:**
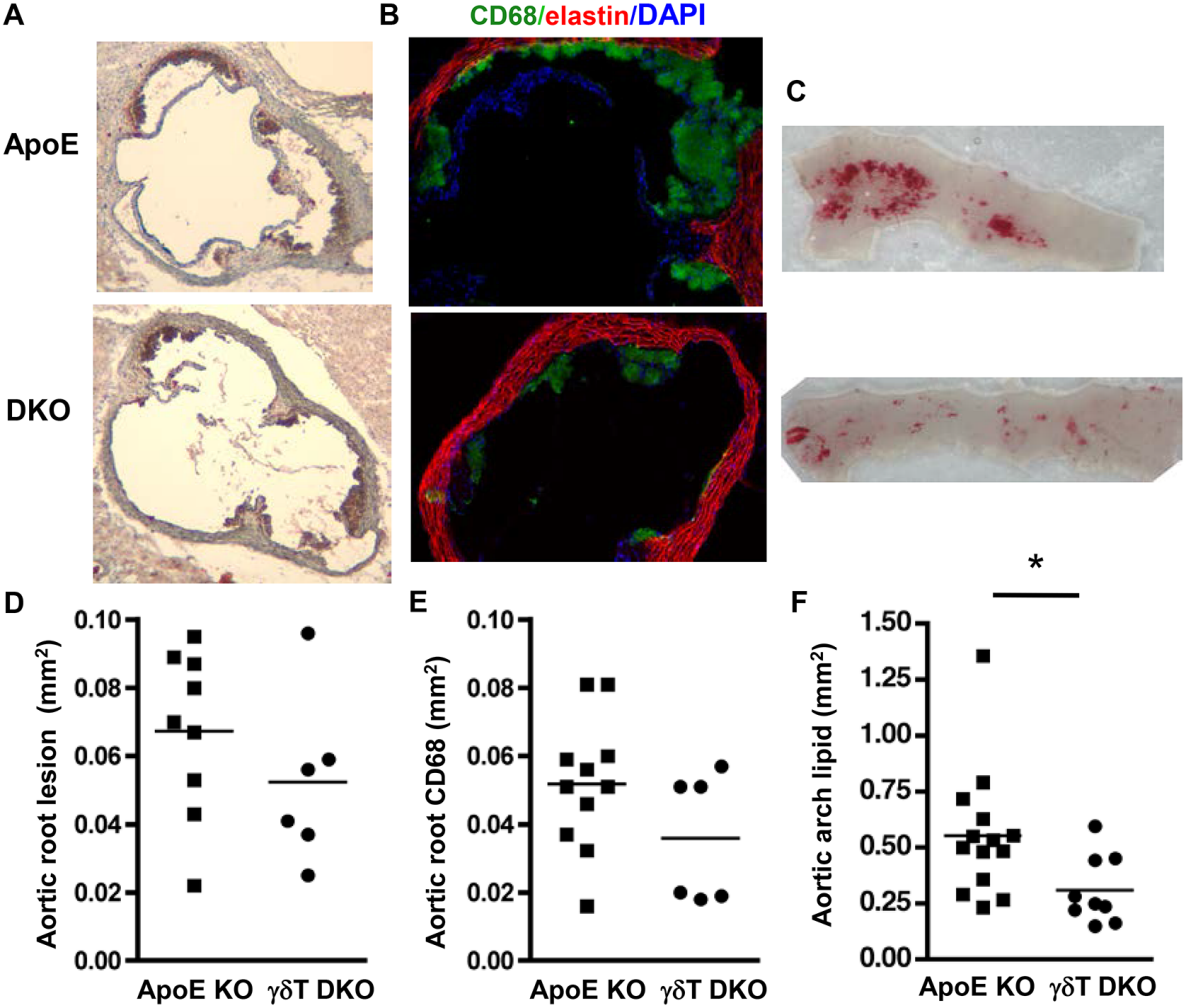
γδT cell deficiency reduces aortic arch lesion area in Western diet-fed ApoE KO mice. Mice were fed Western diet for 4 weeks and aortic root cross sections were stained with **A:** ORO and hematoxylin, or **B:** anti-CD68-Alexa Fluor 488, AlexaFluor 633 to visualize elastin, and DAPI. **C:** Aortic arch segments were stained with ORO *en face.* **D&E:** Charts show total and macrophage-rich lesion areas in the aortic root. The aortic roots of 3 ApoE KO mice and 3 ApoE/γδT DKO mice were excluded from the analysis based on the lack of 3 complete cusps. **F:** Aortic arch lesions were significantly reduced in ApoE/γδT DKO mice (*p<0.03).

Aortic lesions in these mice are shown in Fig. 4 and 5.

When lesion formation was accelerated in younger (14 wk-old) ApoE KO mice by 4 weeks of Western diet, γδT cell-deficiency significantly reduced aortic arch lesion area by 44% (**Fig. 5B** **;** p<0.03). In the aortic root, total, lipid-rich, and macrophage-rich lesion areas were reduced by 22%, 28% and 31%, respectively (**Fig. 5A** and not shown), but statistical significance was not achieved, possibly due to the insufficient number of aortic roots studied due to exclusion based on the absence of 3 complete cusps. Lesion area in the descending aorta was not affected (not shown). γδT cell deficiency did not affect body weight, spleen weight, serum cholesterol, serum triglyceride or serum amyloid A levels in the Western diet-fed mice (**Table 2**). The results suggest that γδT cells promote Western diet-accelerated lesion development in the aortic arch of ApoE KO mice via lipid-independent mechanisms.

### γδT cell deficiency reduces Western diet-induced neutrophilia and lymphopenia, but not inflammatory monocytosis in the blood of ApoE KO mice

**Figure 6:**
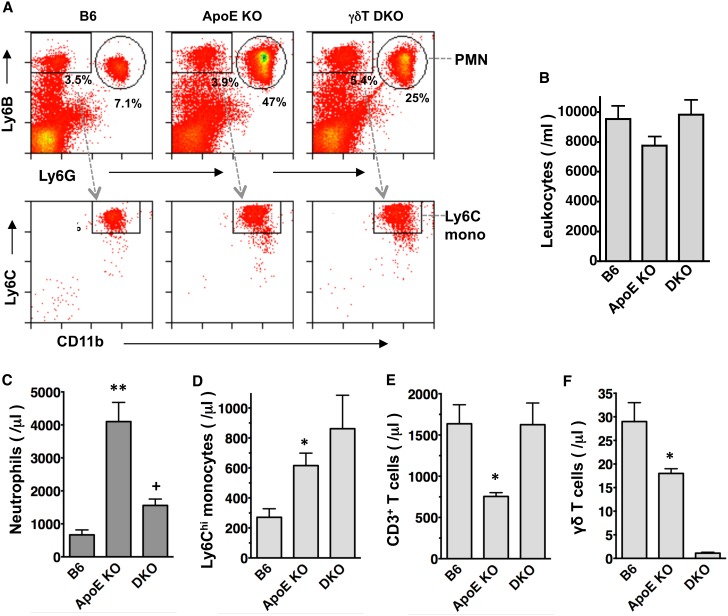
γδT cell deficiency reduces neutrophilia and lymphopenia in ApoE KO mice. Blood leukocytes were stained with anti-Ly6G-FITC, anti-Ly6B(7/4)-PE, anti-CD11b-PERCP Cy5.5 and anti-Ly6C-APC. **A:** Representative FACS plots; X and Y axes are log fluorescence intensity, as in Figs 1 & 3. Neutrophils are Ly6B**^hi^** Ly6G**^hi^**(***top***); inflammatory monocytes are Ly6G^−^, Ly6B**^hi^**, CD11b^+^, Ly6C**^hi^** (***bottom***). Blood neutrophils and Ly6C**^hi^** monocytes are increased in ApoE KO (n = 14) *vs.* B6 (n = 6) mice; γδT cell deficiency (n = 9) reduces neutrophilia (**C**) but not Ly6C**^hi^** monocyte levels (**D**). Circulating CD3^+^ T cells are decreased in ApoE KO, but not ApoE/γδT DKO, *vs.* B6 mice (**E**). γδT cells are decreased in ApoE KO *vs.* B6 mice, and absent in ApoE/γδT DKO mice. *p<0.05, **p<0.01 *vs.* B6; + p<0.05 *vs.* ApoE KO.

Hypercholesterolemia induces neutrophilia and elevates circulating inflammatory monocyte levels, and both of these systemic responses promote aortic lesion progression [34], [35]. We used FACS analysis (**Fig. 6A**) to test whether consuming Western diet for 4 wks induces neutrophilia or inflammatory monocytosis in ApoE KO mice, and whether γδT cell deficiency affects such responses. While total blood leukocyte levels were unchanged in ApoE KO or ApoE/γδT DKO mice (**Fig. 6B**), circulating neutrophils (Ly6G**^hi^**, Ly6B**^hi^**) and inflammatory monocytes (Ly6G^−^, Ly6C**^hi^**, Ly6B**^hi^**, CD11b^+^ [29], [30]) were significantly increased in Western diet-fed ApoE KO mice *vs.* B6 mice, consistent with earlier findings [34], [35]. Importantly, γδT cell deficiency markedly reduced neutrophilia (**Fig. 6A, C**) but did not alter inflammatory monocytosis **Fig. 6A, D**), supporting a specific role of γδT cells in mediating Western diet-induced neutrophilia. Interestingly, total T cell and γδT cell counts were significantly reduced in the blood of ApoE KO mice, while total T cell counts were unchanged in ApoE/γδT DKO mice (**Fig. 6E, F**), possibly due to differential sequestration or homing of T cells in these mice.

### γδT cell deficiency does not affect splenic Th1 and Th17 cell expansion in Western diet-fed ApoE KO mice

**Figure 7:**
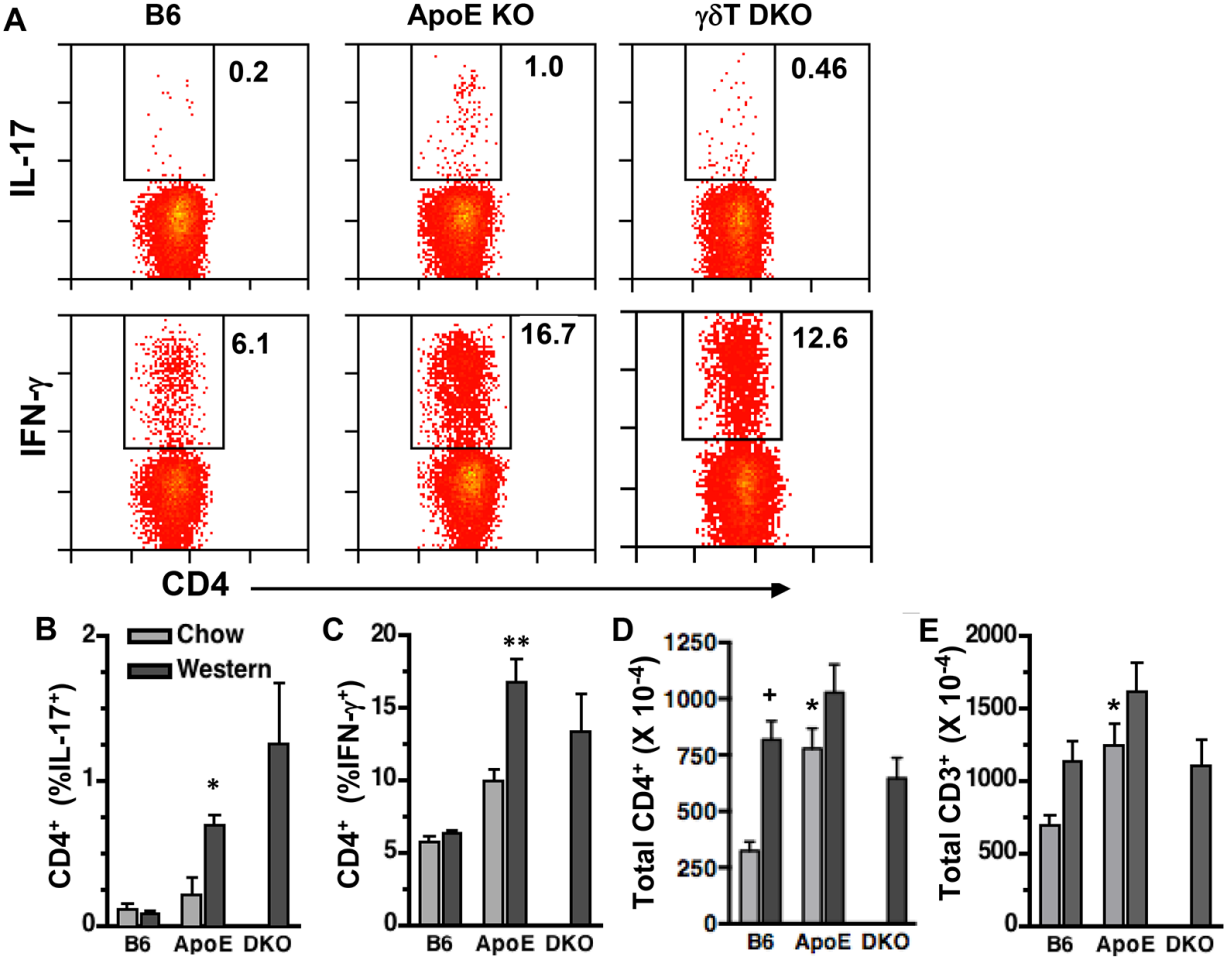
γδT cell deficiency does not affect splenic Th1 or Th17 cell expansion in Western diet-fed ApoE KO mice. Splenocytes of 14 wk-old mice were analyzed by FACS after staining for cell surface CD4 and intracellular IL-17 or IFN-γ. **A:** Representative FACS plots of Western diet fed mice; X and Y axes are log fluorescence intensity, as in Figs 1 & 3. **B–E:** Quantitation for chow- and Western diet-fed mice. **B&C:** IL-17 and IFN-γ expression are increased in CD4^+^ T cells of Western diet-fed ApoE KO (n = 14) *vs.* B6 mice (n = 9), and are similar in ApoE *vs.* γδT DKO (n = 4) mice. **D&E:** Total splenic CD4^+^ and CD3^+^ T cells were increased in chow-fed ApoE KO *vs.* diet-matched B6 mice. *p<0.05 *vs.* diet-matched B6. + p<0.05 *vs.* chow-fed B6.

γδT cells can exert pathogenic effects by promoting Th1 responses in virus-infected mice [36] and Th17 differentiation in mouse models of colitis and multiple sclerosis [37], [38]. To determine whether γδT cells affect Th cell expansion in Western diet-fed ApoE KO mice, we compared splenic Th1 and Th17 cell numbers in wild-type, ApoE KO, and ApoE/γδT DKO mice, using intracellular FACS analysis. We observed that Western diet feeding induced significant increases in both Th1 and Th17 cells in ApoE KO but not B6 mice, due to increased % of CD4^+^ cells expressing IFN-γ and IL-17, respectively (**Fig. 7B&C**). Total splenic CD4^+^ and CD3^+^ T cell numbers were similar in ApoE KO *vs.* B6 mice fed Western diet, although the numbers were increased compared to chow-fed B6 mice (**Fig. 7D&E**). γδT cell deficiency did not significantly reduce either splenic Th1 or Th17 cell expansion in Western diet-fed ApoE KO mice (**Fig. 7**), suggesting that γδT cells are not critical to these responses.

## Discussion

The roles of certain T cell subsets, including Th1, Th17, and Treg in atherogenesis have been extensively studied and defined. Here we show a substantial early pro-atherogenic role of a relatively obscure γδT cell subtype. Our studies focused on early stage atherosclerosis because of the increased prevalence of γδT cells in early stage human lesions [18], [19]. In ApoE KO mice, lesions first develop in the aortic root and arch when consuming a low cholesterol diet, and lesion formation is accelerated at these sites by feeding a high-cholesterol Western-type diet. We found that γδT cells, which constitute only a small fraction of T cells at non-epithelial sites, are the dominant T cell subset in early aortic root and arch lesions in ApoE KO mice. We also found that γδT cell deficiency substantially reduces aortic arch lesion size in Western diet-fed mice, and aortic root lesion size in chow-fed mice, strongly supporting a pathogenic role of γδT cells in the development of nascent early lesions. A recent study of γδT cell-deficient ApoE KO mice fed Western diet for 10 weeks found no effect on total aortic lesion burden, which included the arch and descending aorta but not the aortic root [39]. It will be important in future studies to determine whether the pro-atherogenic effect of γδT cells is specific to the aortic root and arch, as site-specific effects on atherogenesis are well-documented [40]. Alternatively, the proatherogenic effect of γδT cells may become redundant, as additional cellular players become involved after months of severe hypercholesterolemia, or may be counteracted by regulatory, immune-dampening functions of γδT lymphocytes [41]–[43].

Our confocal *en face* analysis is the first, to our knowledge, to precisely analyze γδT cell infiltration into atherosclerotic plaque. The results clearly show that γδT cells, though rare in the intima of wild-type mice, infiltrate early intimal lesions in the aortic root and arch of ApoE KO mice, where they are the dominant T cell subset seen. These findings suggest that this innate T cell is activated locally and/or exerts proatherogenic effects by acting locally within developing lesions. We found γδT cells along the edge of early lesions, where T cells have been shown to reside [44], and where they were much more abundant than CD4^+^ T cells during the early stage of disease. In human atherosclerosis, γδT cells are enriched in fatty streaks and the transition zone between normal intima and atherosclerotic plaque [18], [19], particularly in the earliest stage lesions containing relatively few CD3^+^ T cells. In contrast, γδT cells are rarely seen in the normal intima of children [20]. It will be important in future studies to determine whether γδT cells also contribute to early-stage atherogenesis in humans.

γδT lymphocytes were also the most prevalent T lymphocyte in the adventitia at lesion-prone sites in ApoE-deficient mice, and they were found only in the adventitia, predominantly near the aortic root, in wild-type mice. During advanced disease in humans and mice, other leukocyte classes, including T and B cells, dendritic cells, and macrophages, also infiltrate the adventitia adjacent to atherosclerotic lesions [45]–[47], and they can form highly-organized cell clusters in mice, which may be loci of antigen presentation and B cell selection and maturation during advanced disease [45]. Less well-organized adventitial leukocyte clusters surrounding earlier lesions have likewise been proposed to promote intimal inflammation via unknown mechanisms [48]. It will thus be important to determine whether adventitial *vs.* intimal γδT cells have different roles in atherosclerosis or other vascular diseases.

Our findings suggest that IL-17 may contribute to the proatherogenic role of γδT cells, as many aortic γδT cells expressed IL-17, but not IFN-γ. Also, IL-17, but not IFN-γ, expression was markedly increased in splenic γδT cells of ApoE KO mice *vs.* wild-type mice fed the same high-fat/cholesterol diet, and this expression was highly correlated with aortic arch lesion size in individual ApoE KO mice. Several lines of evidence support an early pro-atherogenic role of IL-17 in ApoE KO mice, as administering it for 5 wks increased early aortic root lesions, whereas inhibiting IL-17 expression or signaling reduced aortic lesion formation and leukocyte accumulation [10]–[12]. However, the proatherogenic role of IL-17 is somewhat controversial because contrasting protective roles of IL-17 have been found in some models [49]. It is possible that IL-17 has distinct, protective roles in later stages of atherosclerosis. For example, IL-17 inhibited atherogenesis in ApoE KO mice fed diets supplemented with very high cholesterol [50], [51] (1.25% *vs*. 0.2% in Western diet), suggesting that distinct mechanisms drive robust lesion progression elicited by excessively high serum cholesterol levels. Also, IL-17 may be protective in later stages of murine and human atherosclerosis by expanding the collagen-rich fibrous cap that covers the lipid core of advanced lesions and promoting lesion stability [52]. Further studies are needed to determine the respective roles of IL-17 derived from CD4^+^ *vs.* γδT cells in early atherogenesis, and whether the IL-17-expressing cells found in human lesions [53] are γδT cells.

γδT cells typically reside at epithelial surfaces in the intestinal mucosa, lung, and skin, serving as “sentinels” that respond rapidly to microbial antigens [17]. Within hours of bacterial or fungal infection, or epithelial cell injury, they produce IL-17, antimicrobial defensins, and chemokines that recruit leukocytes to the site of infection, thereby contributing to host defense [54]. Epithelial γδT cells that produce IL-17 chronically can become pathogenic, as occurs in mouse models of colitis [38] and psoriasis-like dermatitis [55]. γδT17 cells can also promote multiple sclerosis-like autoimmunity in brain and spinal cord [37], [56], wherein they are rare in healthy animals. Our results strongly suggest that γδT cells infiltrate aortic adventitia in healthy mice, but in the setting of ApoE deficiency and its associated hypercholesterolemia they are recruited to the aortic intima, where they can become activated and/or promote local inflammation. It will be important to determine the role of γδT, as well as γδT17, cells in other diseases states involving chronic vascular inflammation.

Our findings confirm that Western diet feeding increases the circulating levels of neutrophils [34] and pro-inflammatory Ly6C^hi^ monocytes [35] in ApoE KO mice and reveal that γδT cells promote neutrophilia, but not inflammatory monocytosis. Our working hypothesis is that γδT cell-derived IL-17 drives neutrophilia in Western diet-fed ApoE KO mice, and that neutrophils in turn contribute to the proatherogenic effects of γδT cells. This hypothesis is supported by findings that IL-17 induces neutrophilia when expressed in wild-type mice [57], [58]. Also, the level of circulating neutrophils correlates positively with the extent of early aortic lesion progression in the aortic root of ApoE KO mice, and neutrophil depletion reduces lesion progression [34], [59]. Neutrophils are thought to exacerbate atherosclerosis via multiple mechanisms, including release of reactive oxygen species and granule proteins, such as CRAMP and azurocidin, which can promote activation and recruitment of inflammatory monocytes to aortic plaque [60]–[62]. Elucidating the connections between hypercholesterolemia-activated γδT cells, IL-17, neutrophilia and lesion progression is a key future goal.

We focused our study on early disease in order to uncover early, potentially targetable atherogenic mechanisms. Evidence from mice shows that intervening early by lowering plasma cholesterol can reduce lesion burden, whereas once lesions are more advanced it is difficult to reverse atherosclerosis [63]. Also, our studies did not invoke prolonged and severe hypocholesterolemia, which may engage multiple redundant and overwhelming inflammatory pathways that do not reflect human disease. During early disease in mice, lesions forming in the lesion-prone aortic root and arch consist primarily of focal accumulation of lipid-laden foam cells, and it has long been known that T cells are present in lesions, particularly at early stages [64]. Here we report that the majority of T cells found in early atherosclerotic lesions in mice are γδT cells, pointing to the necessity of using TCR subset-specific antibodies to characterize the T cell subset associated with vascular tissue in murine and human atherosclerosis and other vascular diseases. We also report that γδT cells promote lesion progression during the early development stage. Elucidating pathogenic roles of γδT cells in human atherosclerosis may provide a basis for targeting them as a novel approach to therapeutic intervention in vascular disease.

## Supporting Information

Figure S1 **FACS analysis validation. A:** γδT cells are absent in ApoE/TCRδ DKO mice, shown by staining peripheral lymph node cells with anti-CD3-FITC and TCRγδ-APC. **B:** Specificity of CD45 and CD3 staining in aortic cells from ApoE KO mouse aorta. Cells were stained with anti-CD3-FITC, anti-CD45-PE, or appropriate isotype control IgG, as indicated. CD45^+^ and CD3^+^ populations are distinct. **C:** Specificity of staining for intracellular IL-17 and IFN-γ. Splenocytes were stained with cell surface marker antibodies (CD3, CD4, TCRγδ) then fixed, permeabilized and incubated with appropriate isotype IgG.
(TIF)

Figure S2 **Immunostaining validation.** Aortic root adventitia of a Western diet-fed ApoE KO mouse stained with anti-CD3-FITC (*green*), anti-TCRγδ -APC (*red*), and DAPI (*blue*) Staining is specific, and readily distinguishes conventional αβ (arrows) *vs.* γδT cells (arrowheads).
(TIF)

Figure S3 **γδT cells are more prevalent than CD4^+^ T cells in early lesions and do not themselves express CD4^+^.** *En face* confocal images of early aortic root lesions of ApoE KO mice (14 wk-old, fed Western diet for 4 wks; n = 3) stained with anti-CD4-Alexa Fluor 488 (*green*), anti-TCRγδ APC (*red*), and DAPI (*blue*).
(TIF)

Figure S4 **CD4^+^ T cells are more prevalent in advanced lesions.** Aortic root from aged chow-fed mice (10–11 mos, n = 3) were stained with CD3-FITC, anti-CD4 APC and DAPI. CD3^+^CD4^+^ T cells are more prevalent (63% of total CD3^+^ T cells) along the edge of advanced aortic root lesions with necrotic cores compared to early lesions seen in Fig. S3.
(TIF)

Figure S5 **Splenic γδT17 cells: correlation analysis.** Aortic arch lesion area is correlated with IL-17 expression in γδT cells of individual ApoE KO mice fed Western diet for 4 wks (**A**). Total splenic γδT17 cells were also significantly and positively correlated with total splenic Th17 cells (**B**), negatively correlated with serum cholesterol levels (**C**), and not correlated with serum triglyceride levels (**D**).
(TIF)
