# Supplementary figures and images for: γδT Cells Are Prevalent in the Proximal Aorta and Drive Nascent Atherosclerotic Lesion Progression and Neutrophilia in Hypercholesterolemic Mice
Source: PLoS One. 2014 Oct 14;9(10):e109416. doi: 10.1371/journal.pone.0109416 (PMC4196850; doi:10.1371/journal.pone.0109416)

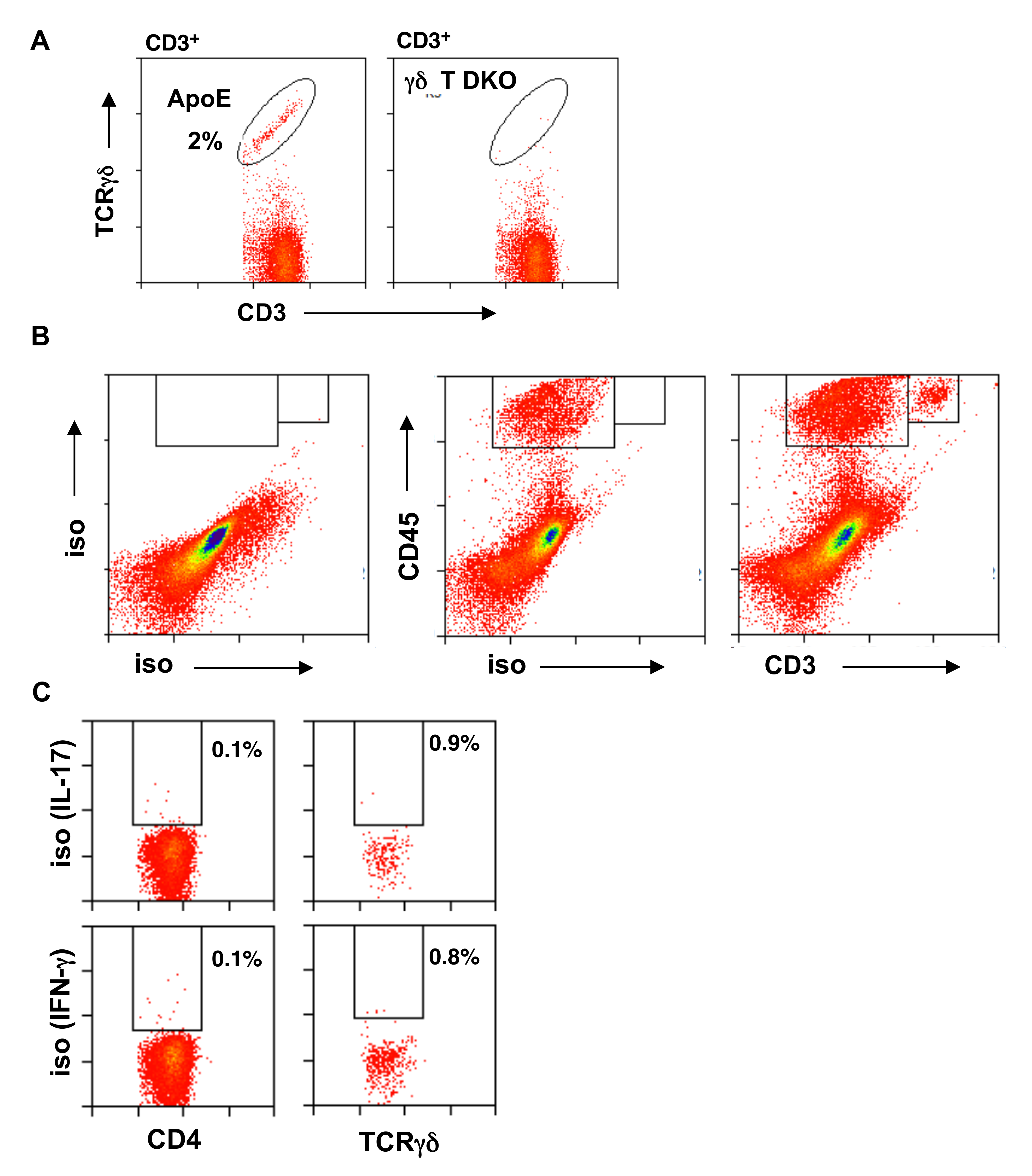

Supplement: Figure S1 — FACS analysis validation. A: γδT cells are absent in ApoE/TCRδ DKO mice, shown by staining peripheral lymph node cells with anti-CD3-FITC and TCRγδ-APC. B: Specificity of CD45 and CD3 staining in aortic cells from ApoE KO mouse aorta. Cells were stained with anti-CD3-FITC, anti-CD45-PE, or appropriate isotype control IgG, as indicated. CD45+ and CD3+ populations are distinct. C: Specificity of staining for intracellular IL-17 and IFN-γ. Splenocytes were stained with cell surface marker antibodies (CD3, CD4, TCRγδ) then fixed, permeabilized and incubated with appropriate isotype IgG. (TIF) [file pone.0109416.s001.tif]

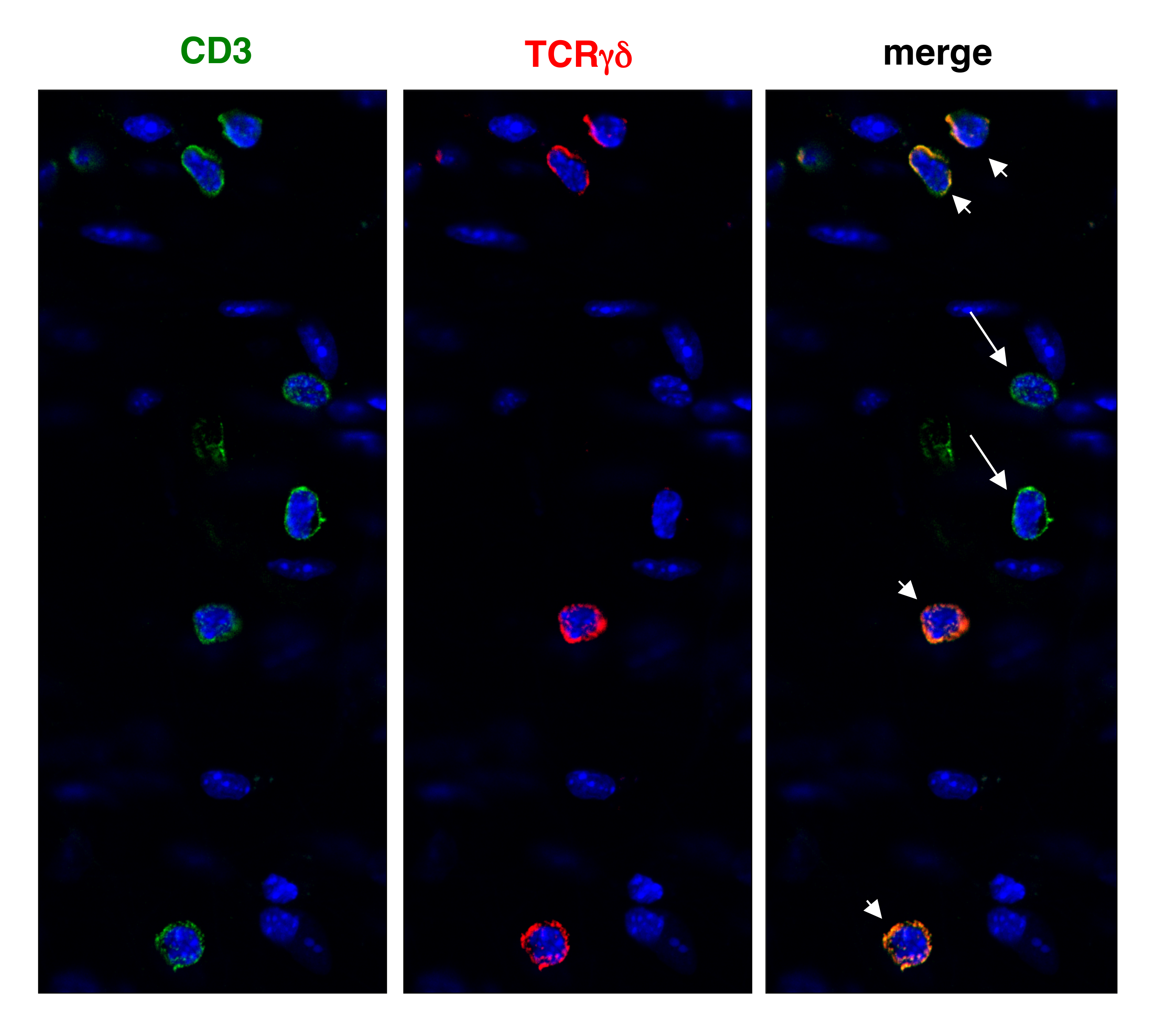

Supplement: Figure S2 — Immunostaining validation. Aortic root adventitia of a Western diet-fed ApoE KO mouse stained with anti-CD3-FITC (green), anti-TCRγδ -APC (red), and DAPI (blue) Staining is specific, and readily distinguishes conventional αβ (arrows) vs. γδT cells (arrowheads). (TIF) [file pone.0109416.s002.tif]

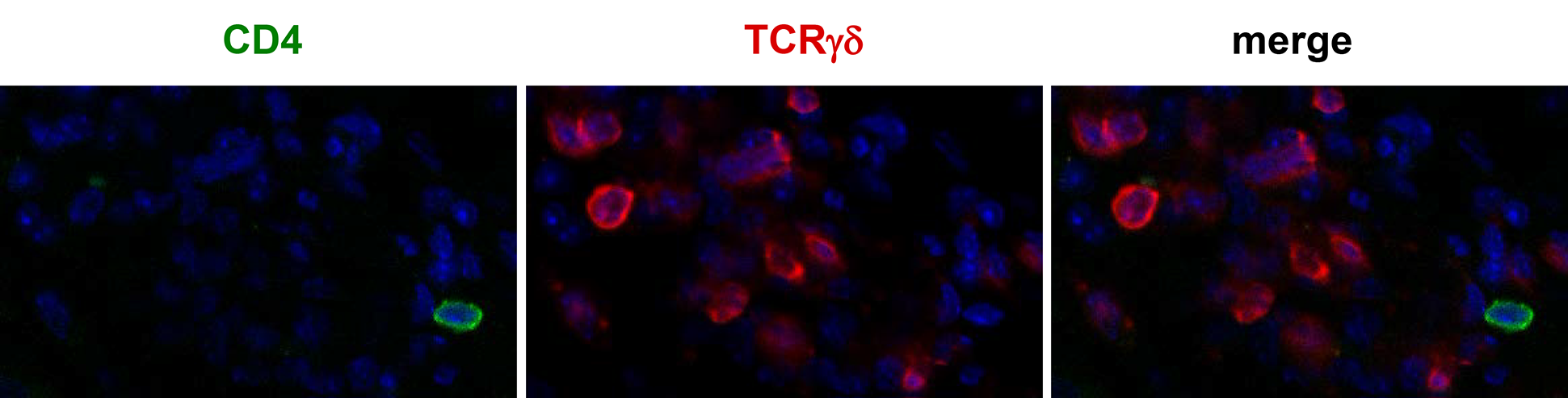

Supplement: Figure S3 — γδT cells are more prevalent than CD4+ T cells in early lesions and do not themselves express CD4+. En face confocal images of early aortic root lesions of ApoE KO mice (14 wk-old, fed Western diet for 4 wks; n = 3) stained with anti-CD4-Alexa Fluor 488 (green), anti-TCRγδ APC (red), and DAPI (blue). (TIF) [file pone.0109416.s003.tif]

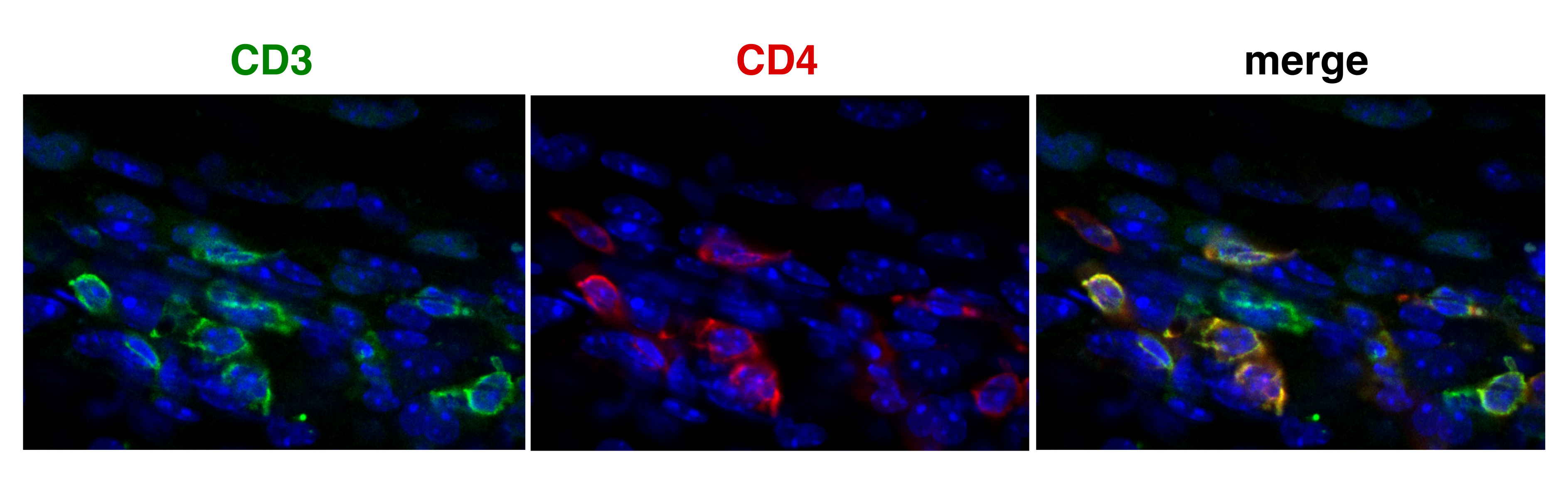

Supplement: Figure S4 — CD4+ T cells are more prevalent in advanced lesions. Aortic root from aged chow-fed mice (10–11 mos, n = 3) were stained with CD3-FITC, anti-CD4 APC and DAPI. CD3+CD4+ T cells are more prevalent (63% of total CD3+ T cells) along the edge of advanced aortic root lesions with necrotic cores compared to early lesions seen in Fig. S3. (TIF) [file pone.0109416.s004.tif]

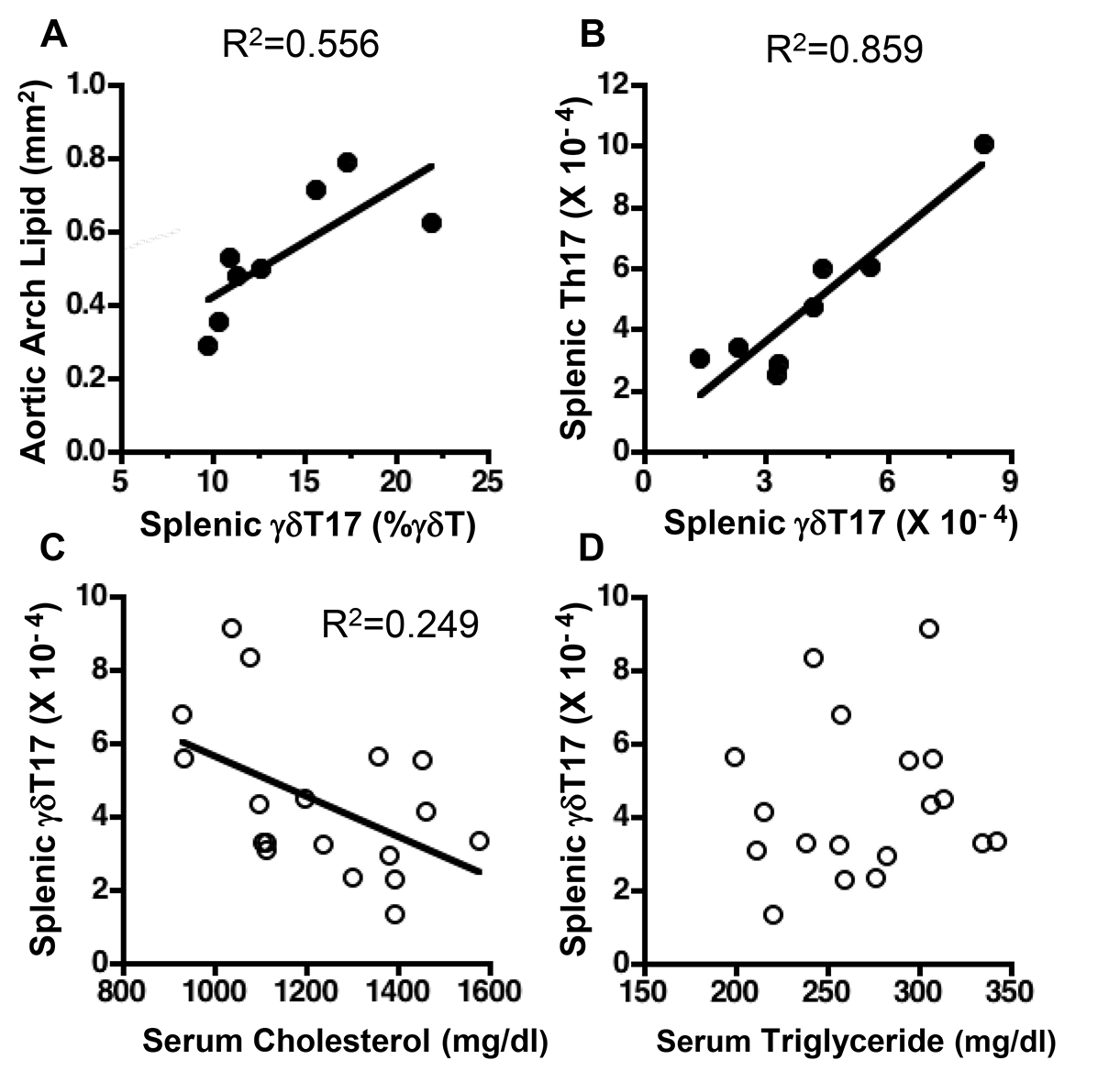

Supplement: Figure S5 — Splenic γδT17 cells: correlation analysis. Aortic arch lesion area is correlated with IL-17 expression in γδT cells of individual ApoE KO mice fed Western diet for 4 wks (A). Total splenic γδT17 cells were also significantly and positively correlated with total splenic Th17 cells (B), negatively correlated with serum cholesterol levels (C), and not correlated with serum triglyceride levels (D). (TIF) [file pone.0109416.s005.tif]
